# Influence of Cold Pre-Fermentation Maceration on the Volatilomic Pattern and Aroma of White Wines

**DOI:** 10.3390/foods12061135

**Published:** 2023-03-08

**Authors:** Laura Alti-Palacios, Juana Martínez, José A. C. Teixeira, José S. Câmara, Rosa Perestrelo

**Affiliations:** 1 Instituto de Ciencias de la Vid y el Vino (Gobierno de La Rioja, Universidad de La Rioja, CSIC, Finca La Grajera) Ctra. de Burgos, Km. 6, 26007 Logroño, Spain; 2 Departamento de Engenharia Biológica, Universidade do Minho, 4710-057 Braga, Portugal; 3 CQM—Centro de Química da Madeira, Universidade da Madeira, Campus da Penteada, 9020-105 Funchal, Portugal; 4 Departamento de Química, Faculdade de Ciências Exatas e Engenharia, Universidade da Madeira, Campus da Penteada, 9020-105 Funchal, Portugal

**Keywords:** cold pre-fermentation maceration, aroma compounds, white wines, HS-SPME/GC-MS, statistical analysis

## Abstract

Aroma compounds play a key role in wine quality due to their importance in wine aroma. The aim of the present study is to investigate the influence of cold pre-fermentative maceration (CPM) treatment on aromatic and sensory properties of white wines from four grape varieties (Tempranillo Blanco, Maturana Blanca, Viura and Garnacha Blanca) during two consecutive years (2019 and 2020). A total of 62 aroma compounds belonging to different chemical families were identified using headspace solid-phase microextraction combined with gas chromatography mass spectrometry (HS-SPME/GC-MS). CPM treatment enhanced the total relative concentration of alcohols, esters and acids compared to control wines. Regarding sensorial properties, esters made the greatest contribution to the studied white wines, mainly through the development of floral and fruity notes. On the other hand, CPM treatment did not significantly influence the total relative concentration of terpenoids, and different trends were observed according to grape variety and vintage. The obtained results showed differences in the wine’s aromatic complexity according to the grape variety, the vintage and the treatment applied and suggested that CPM treatment could represent a suitable approach to manipulate the aromatic profile and enhance the aromatic quality and complexity of wine.

## 1. Introduction

Wine aroma is a crucial criterion in determining wine quality and is influenced by volatile secondary metabolites belonging to different chemical families, including alcohols, esters, acids, carbonyl compounds and terpenoids, among others [1]. The contribution of these volatiles is not the same for wine aroma, which depends on their concentration and odor threshold (OT), matrix effects, interaction effects and intermodal interaction effects, among others [2]. However, the growing prerequisites of consumers for distinctive aromatic properties encourage winemakers to develop a diversity of approaches to manipulate particular aroma compounds and enhance the complexity of wines [3]. In this sense, cold pre-fermentation maceration (CPM) is a popular winemaking technology frequently applied in the production of white wines prior to alcoholic fermentation to enhance aromatic intensity and complexity, especially in white wines, further improving their varietal character, stability and color intensity [3,4,5,6]. This vinification technique typically involves the application of low-temperature maceration (5–15 °C) of the must, skins and seeds for a period of 4–10 days [4]. It is generally performed with dry ice (solid dioxide carbon (CO_2_)), which produces a thermal shock that causes the cells of the skins to break, increasing their contact with the must, which increases the extraction of aromatic and phenolic compounds from grape berries and their solubilization into must and wines [4,7]. The CPM technique can change the content of nutrients released in grape must, such as nitrogen sources, vitamins and fatty acids, consequently affecting cell growth and the formation of aroma compounds [3].

The results obtained from several investigations evaluating the influence of the CMP technique on wine quality are highly variable and depend on the characteristics of the grape (e.g., variety and degree of maturity), the maceration conditions (e.g., time and temperature) and the cooling mode (e.g., grape freezing, liquid nitrogen (N_2_), dry ice or heat exchangers) [1,4,5]. Most previous CPM investigations have focused on the evaluation of the effect of this vinification technique on wine color and on the phenolic pattern [2,8,9]. On the other hand, few studies have been reported related to the influence of CPM on the aroma composition of wines. Petrozziello et al. [6] observed that fruity and floral sensory descriptors were improved in wine aroma after application of the CPM technique, but no significant differences in volatile composition were observed between control and treated wines. A more recent study on the influence of CPM treatment on the volatile composition of wines of Cabernet Sauvignon fermented in fermenters of different scales, namely automatic pumping-over (PO) and automatic punching-down (PD) tanks [4]. The obtained data demonstrated that a PO tank is more effective compared to a PD tank. Luan et al. [3] evaluated the effects of different durations of the CPM technique on the volatile aroma of *Saccharomyces cerevisiae* cofermentation with *Hanseniaspora opuntiae* or *Pichia kudriavzevii*. The results demonstrated that the duration of CPM strongly affected the formation of aroma compounds, and clear species-dependent differences were verified. Lukić et al. [7] studied the effect of six maceration treatments on the volatile aroma and polyphenol composition of red wines and found that CPM resulted in the lowest extraction of seed tannins the and highest extraction of acetaldehyde, ethyl acetate, C6 compounds and esters compared to the control.

Considering the importance of volatile aroma with respect to the final quality of wines, the objective of the current work was to evaluate the influence of CPM with dry ice (solid carbon dioxide (CO_2_)) on the aromatic quality of white wines produced using four white varieties of *Vitis vinifera* L. authorized according to the Rioja Qualified Designation of Origin (D.O.Ca. Rioja), namely *V. vinifera* ‘Tempranillo Blanco’ (TB), *V. vinifera* ‘Maturana Blanca’ (MB), *V. vinifera* ‘Garnacha Blanca’ (GB) and *V. vinifera* ‘Viura’ (V). ‘Tempranillo Blanco’ and ‘Maturana Blanca’ are autochthonous varieties of the D.O.Ca. Rioja. ‘Tempranillo Blanco’ comes from a natural genetic mutation [10] found in a single cane of a red Tempranillo vine discovered in an old vineyard in Murillo de Río Leza (La Rioja) in 1988. It does not exist anywhere else in the world. Maturana Blanca is the variety that occupies the least surface area among authorized grapes, but its plantation is currently recovering. It is the oldest grape variety to have a written record in Rioja. ‘Garnacha Blanca’ is a variety with a small surface area among authorized in the D.O.Ca. Rioja grapes, but its plantation currently is increasing. Viura is the main white grape variety grown in the D.O.Ca. Rioja (Consejo Regulador de la Denominación de Origen Calificada Rioja) [11]. The volatile aroma was established using the headspace solid-phase microextraction combined gas chromatography mass spectrometry (HS-SPME/GC-MS) methodology. This methodology allows for evaluation of the diversity and similarity of the volatile aroma of white wines obtained using the traditional white winemaking process (control wines, C) and CPM wines. A combination of a chromatographic dataset with multivariate statistical data analysis (MSDA) was also used to extract useful information concerning the enrichment of volatile aroma using the CPM technique.

## 2. Materials and Methods

### 2.1. Chemicals and Reagents

Sodium chloride (99.5%, foodstuff grade) and 3-octanol (used as internal standard (IS), 99 %) were obtained from Sigma-Aldrich (Madrid, Spain), whereas the GC carrier gas, helium, of purity 5.0 was obtained from Air Liquide (Lisbon, Portugal). The SPME holder for manual sampling, glass vials and fiber were purchased from Supelco (Aldrich, Bellefonte, PA, USA). The SPME device included a fused silica fiber coating partially cross-inked with 50/30 µm divinylbenzene/carboxen/polydimethylsiloxane (DVB/CAR/PDMS). Prior to use, the SPME fiber was conditioned at 270 °C for 30 min in a GC injector according to the manufacturer’s recommendations. Then, the fiber was conditioned daily for 10 min at 250 °C. The alkane series, C8 to C20, with a concentration of 40 mg/L in *n*-hexane used to calculate the retention index (RI) was supplied by Fluka (Buchs, Switzerland). Ultrapure water was obtained from a Milli-Q^®^ system (Millipore, Bedford, MA, USA).

### 2.2. White Wines

Healthy mature-state white *V. vinifera* L. grape varieties, namely ‘Tempranillo Blanco’ (TB), ‘Maturana Blanca’ (MB), ‘Viura’ (V) and ‘Garnacha Blanca’ (GB), from the 2019 and 2020 harvest were collected in a good sanitary state and optimal maturity state based on maximum sugar content (soluble solids: 21.5–23.5° Brix). The experiment was carried out in an experimental vineyard located in La Grajera (42°26′21″ N, 2°30′49″ W), Logroño (Spain), planted in 2007 in a 2.90 × 1.10 m framework trellis system, double Royat cordon training system (2.90 × 1.10 m) and a statistical design of random blocks with three repetitions of thirty vines per variety. All vinifications were conducted in the experimental winery of the Institute of Grapevine and Wine Sciences.

The C wines were prepared using the traditional white winemaking process. The grapes were destemmed and crushed. The must was separated from the skins using a pneumatic press (3 bars). The obtained must was sulphited (60 mg/L) and racked with pectolytic enzymes (1 g/hL Lafazym CL, Laffort S.L.) and stored in a cold room (15 °C) for 15 h. Then, all vinifications were carried out in triplicate in 30 L stainless steel tanks. The must was inoculated with 20 g/hL of commercial yeast (Zymaflore X16, Laffort S.L.) to start the fermentation process, which was controlled at 18–20 °C in a room equipped with a temperature control system.

For CPM wines, the grapes were destemmed, crushed and mashing with dry ice for 6 h to 13 ± 1 °C. Then, the must was separated from the skins using a pneumatic press (3 bars). The subsequent winemaking process of the CPM wines was the same as for C wines.

Every day, a temperature and density control test of the wines was carried out. The alcoholic fermentation was finished when the concentration of reducing sugars was less than 2 g/L and white wines were dry. Then, the wines were racked, sulphited (40 mg/L) and stabilized at 5 °C for one month. After that, two vinification process were evaluated and compared: one for the control wines (C) and other for cold pre-fermentative maceration wines (CPM).

### 2.3. Oenological Parameters

The studied oenological parameters in white wines were determined based on official analysis methods [12]: pH, total acidity (expressed as g/L tartaric acid) and alcohol degree (% vol: mL ethanol/100 mL wine). The methods applied to assess these parameters are accredited by ISO 17025 norm, and the uncertainty was also stipulated based on this norm.

Total phenolics were determined as total polyphenol index (TPI) by spectrophotometric absorbance (A) at 280 nm according to Ribéreau et al. [13] after wine centrifugation (2500× *g* for 10 min) at 10 °C to remove any particulate matter and diluted with distilled water (1:10). Absorbance measurements were conducted in quartz cuvettes with a 1 mm optical path. The total content of hydroxycinnamic acids (HCAs) was determined by spectrophotometry as chlorogenic acid equivalents at A_327 nm_, following the method described by Dumitru Gabriela [14]. Absorbance measurements were conducted in quartz cuvettes with a 1 mm optical path. Yellow color was determined by spectrophotometric analysis at A_420 nm_ according to Dumitru Gabriela [14]. Absorbance measurements were conducted in glass cuvettes with a 1 cm optical path. Absorbance measurements were conducted in a Helios Omega spectrophotometer (Thermo Fisher Scientific, Waltham, MA, USA).

### 2.4. HS-SPME Procedure

The HS-SPME experimental parameters were previously established [15]. Briefly, 4 mL of wine, 0.5 g of NaCl (to promote the “salting-out” effect by decreasing the solubility of volatile aromas in the water-based phase) and 10 µL of 3-octanol (IS, 252 µg/L) were placed into a 20 mL amber headspace glass vial containing a magnetic stirring bar (0.5 × 1 cm). The vial was capped with a polytetrafluoroethylene (PTFE) septum and placed in a thermostatic bath at 40 ± 1 °C under constant magnetic stirring (400 rpm). Then, the SPME fiber (DVB/CAR/PDMS) was exposed to the sample’s headspace for 45 min. Finally, the fiber was withdrawn into the holder needle, removed from the vial and directly inserted into GC injector to promote the thermally desorption of volatile aromas extracted on the fiber. Three independent aliquots of each sample were analyzed in triplicate. SPME fiber was thermally conditioned according to the manufacturer’s instructions before use, and daily conditioning was carried out for 10 min before the first extraction to ensure the absence of carryover. Moreover, blanks corresponding to the analysis of the coating fiber not submitted to any extraction procedure were run between sets of three analyses.

### 2.5. GC-MS Conditions

After the extraction/concentration step, the SPME coating fiber containing the volatile aromas was manually introduced into the GC injection port at 250 °C (equipped with a glass liner, 0.75 mm I.D.) and left for 6 min for desorption. The desorbed volatile aromas were separated in an Agilent Technologies 6890N (Palo Alto, CA, USA) gas chromatography system equipped with a SUPELCOWAX^®^ 10 fused silica capillary column (60 m × 0.25 mm i.d. × 0.25 µm film thickness) supplied by Supelco (Bellefonte, PA, USA) with helium (Helium N60, Air Liquid, Lisbon, Portugal) as carrier gas at a flow rate of 1 mL/min (column-head pressure: 13 psi). The GC oven temperature was programmed as follows: an initial temperature of 40 °C (1 min) ramped at 2.5 °C /min to 220 °C (10 min) for a total GC run time of 83 min. MS detection was performed in full scan mode (30–300 *m*/*z*) in an Agilent 5975 quadrupole inert mass-selective detector (Santa Clara, CA, USA). For the MS system, the temperatures of the transfer line, quadrupole and ionization source were 250, 150 and 230 °C, respectively; electron impact mass spectra were recorded at 70 eV, and the ionization current was about 30 µA. Volatile aroma identification was achieved based on their mass spectra compared with those in the National Institute of Standards and Technology (NIST) MS 05 spectral database (Gaithersburg, MD, USA) with a matching probability ≥80% and by comparing their retention index (RI) calculated using n-alkanes C8–C20 as external references with RI values reported in the literature (LRI) for similar columns [16,17,18]. The aroma compound concentration was estimated as a relative concentration using the added amount of 3-octanol (IS) according to the following equation: aroma compound relative concentration = (aroma compound GC peak area/IS GC peak area) × IS concentration.

### 2.6. Data Treatment and Multivariate Statistical Analysis

Multivariate statistical analysis (MSDA) was performed using the MetaboAnalyst 5.0 web-based tool [19]. The raw GC-MS data were preprocessed to remove aroma compounds with missing values and then normalized (data transformation by cubic root and data scaling by autoscaling). The data matrix was subjected to a one-way analysis of variance (ANOVA) followed by Fisher’s test for post hoc multiple comparisons of means from data of the investigated white wines at a *p*-value < 0.05 to identify significant differences. Principal component analysis (PCA) and partial least squares discriminant analysis (PLS-DA) were used to provide insights into the separations between the two vinification processes, as well as among the investigated white wines. The aroma compounds with variable importance in projection (VIP) scores ≥ 1 and differentially expressed in the univariate analysis were identified as potential candidates for white wine characterization, as well as the vinification process under study. Hierarchical cluster analysis (HCA) was carried out using the most significant aroma compounds identified in white wines obtained by ANOVA and was generated through Ward’s algorithm and Euclidean distance analysis, aiming to identify clustering patterns for the characterization of the analyzed white wines. Significance was established at *p* < 0.05.

## 3. Results and Discussions

### 3.1. Physicochemical Parameters

The oenological parameters (Table 1) show that the total acid content (expressed as g/L of tartaric acid) decreased significantly between C and CPM wines, whereas the remaining oenological parameters increased significantly between C and CPM wines, namely pH, potassium, yellow color (A_420 nm_), total HCA content (A_327 nm_) and total phenolic content (A_280 nm_). Moreover, no significant difference was observed in terms of ethanol content (% *v*/*v*) among C and CPM wines. These varieties were previously characterized in a study by Martínez et al. [20].

### 3.2. Volatile Aroma of White Wines Using HS-SPME/GC-MS Methodology

HS-SPME/GC-MS methodology was used to establish the white wine volatile aroma, as a sensitive analytical approach to explain the unique aroma descriptors of the investigated white wines. Considering the four wine varieties, the influence of the CPM technique and the vintage (2019 or 2020), a set of 62 aroma compounds (Table 2), including 21 esters, 15 alcohols, 17 terpenoids, 7 carbonyl compounds and 2 acids, was identified by matching the obtained mass spectra with the spectra of the reference compounds in the NIST Mass Spectral Search Program with a resemblance percentage above 80% and by comparison of the calculated RIs with the values reported in the literature (LRI) for polyethylene glycol (or equivalent) columns. Moreover, Table 2 reports all aroma compounds identified, as well as their odor sensory descriptors [7,18,21] and odor thresholds (OT) [4,22,23,24,25], whereas Table S1 (Supplementary Material) shows the relative concentration of the identified aroma compounds; some differences were also detected in both the qualitative and semi-quantitative (relative concentration) expressions. Three-quarters of the identified aroma compounds were common to all the investigated control and CPM white wines. On the other hand, some aroma compounds were detected in specific white wines, such as 4-methyl-benzaldehyde, dodecanal, isopulegol and citral in ‘Tempranillo Blanco’; β-pinene in ‘Maturana Blanca’; and nonanal in ‘Garnacha Blanca’.

The distribution of chemical families by white wine, CPM technique and vintage is illustrated in Figure S1 (Supplementary Material), verifying that alcohols represent the largest chemical family in terms of relative concentration of aroma compounds, followed by esters, acids, terpenoids and carbonyl compounds.

**Figure 1:**
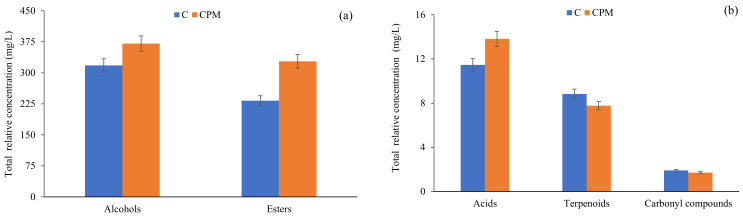
Influence of the CPM technique on the levels of major (**a**) and minor (**b**) chemical families identified in white wines.

Alcohols are secondary products of yeast alcoholic fermentation, and based on their concentration, this chemical family can have either a positive (at concentrations lower than 300 mg/L) or negative (at concentrations higher than 400 mg/L) influence on wine aroma [4]. The total relative concentration of alcohols in ‘Tempranillo Blanco’, ‘Maturana Blanca’, ‘Viura’ and ‘Garnacha Blanca’ wines in all samples analyzed was below 300 mg/L; therefore, it is expected that this chemical family contributes positively to the complexity of wine aroma. 3-Methyl-1-butanol (relative concentration ranging from 29.0 to 55.7 mg/L) and phenylethyl alcohol (2.45 to 6.85 mg/L) were the most abundant alcohols detected in the studied white wines. However, these two alcohols are detected in all white wines at concentrations lower than their OTs; therefore, they were not expected to contribute to the complexity of wine aroma. Moreover, as can be seen in Figure 1, in general, the CPM technique showed a significant effect on the total relative concentration of alcohols, independent of the vintage. On the other hand, the CPM technique applied to white wines obtained from ‘Maturana Blanca’ did not show a significant difference on the total relative concentration of alcohols. This results is in agreement with a recent study that demonstrated that the CPM technique increased the alcohol concentration in Syrah and Monastrell young wines, whereas a decrease in the concentration of this chemical family was observed for Cabernet Sauvignon young wines [1].

**Figure 2:**
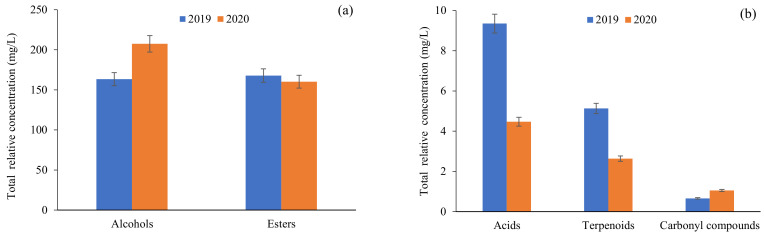
Influence of harvest on total relative concentration (µg/L) of major (**a**) and minor (**b**) chemical families identified in white wines.

Esters (including ethyl esters and acetates) are the second major chemical family in wine and have a remarkable effect on wine aroma complexity, especially ethyl esters, due to their low OT (few µg/L, Table 2). The biosynthesis of esters depends on the grape ripening state, must aeration, yeast strain and fermentation technologies [1,2]. The total relative concentration of esters detected in the studied white wines ranged from 12.1 to 61.5 mg/L and from 17.6 to 76.7 mg/L in wines made without and with the CPM technique, respectively. It was possible to observe that the CPM technique has a positively influence on the total relative concentration of esters (Figure 1). On the other hand, the harvest did not influence the total relative concentration of ethyl esters (Figure 2). This result is in agreement with observations in the production of Sauvignon Blanc [28] and Chardonnay wine [29]. In the current study, four ethyl esters were identified, namely ethyl butanoate (relative concentration ranging from 0.10 to 0.75 mg/L), ethyl hexanoate (1.71 to 8.64 mg/L), ethyl octanoate (5.01 to 35.5 mg/L) and ethyl decanoate (0.11 to 1.43 mg/L), all of which were detected in the investigated white wines, independent of grape variety and vintage, at relative concentrations higher than their OT. Therefore, these ethyl esters might contribute to the complexity of wine aroma, either directly or synergistically, with fruity, sweet and floral sensory descriptors. On the other hand, pentyl propionate and ethyl 5-hexenoate were not detected in ‘Maturana Blanca’ and ‘Viura’ wines from 2020. Acetate esters are generated by the reaction of acetyl-CoA with higher alcohols formed by degradation of amino acids or fatty acids [30]. In the current study, two acetate esters were identified, namely isoamyl acetate (relative concentration ranging from 2.30 to 27.7 mg/L) and 2-phenylethyl acetate (1.02 to 6.32 mg/L), both of which were detected in the investigated white wines, independent of grape variety and vintage, at relative concentrations higher than their OT. These acetate esters contribute to the complexity of wine aroma with fruity notes [31,32].

Acids are produced by yeast and bacteria during fatty acid metabolism. This chemical family can contribute to the complexity of the wine bouquet, even if present at sub-sensory threshold levels, whereas when present at concentrations higher than their OTs, they have a negative effect on wine aroma [4]. As can be observed in Figure 1 and Figure 2, the CPM technique and harvest had a significant influence on the total relative concentration of acids. From a sensorial point of view, hexanoic acid (relative concentration ranging from 0.34 to 0.74 mg/L) and octanoic acid (0.39 to 2.82 mg/L), independent of grape variety and vintage, were detected at relative concentrations lower than their OTs; therefore, no sensorial contribution to wine aroma was expected.

Terpenoids are secondary plant constituents, the biosynthesis of which begins with acetyl-CoA [22] and significantly contributes to the varietal aroma of some aromatic wines due to their low OTs (Table 2). Piperitone was not detected in ‘Maturana Blanca’ wines, whereas β-pinene was only detected in ‘Maturana Blanca’ wines from 2019 submitted to the CPM technique. In addition, 3-carene was only detected in ‘Tempranillo Blanco’ and ‘Maturana Blanca’ wines, independent of the vintage. In general, only linalool and β-myrcene were detected in white wines studied at concentrations above their OTs. However, β-myrcene in ‘Viura’ wines from 2019 (0.01 mg/L) was present at a relative concentration lower than its OT (14 µg/L). The CPM technique influenced the total relative concentration of terpenoids, and different trends were observed depending on the grape variety and vintage. This result was difficult to explain, since terpenoids are influenced by several processes that occur during mash fermentation, namely their extraction from grape skin, hydrolysis of bound forms and conversions induced by yeast, among others factors [7]. In addition, Yilmaztekin et al. [33] verified that increasing the CMP duration did not result in an increase in free terpenes in pure and mixed fermentations. Moreover, Lukić et al. [7] observed that the CPM technique decreased the total amount of terpenoids.

Among the carbonyl compounds identified in the analyzed white wines, only acetaldehyde, octanal and benzaldehyde were common to all. The total relative concentration of carbonyl compounds detected in the studied white wines ranged from 0.13 to 0.38 mg/L and from 0.12 to 0.29 mg/L in wines made without and with CPM treatment, respectively. It was possible to observe that CPM treatment slightly decrease the total relative concentration of carbonyl compounds.

### 3.3. Multivariate Statistical Analysis

**Figure 3:**
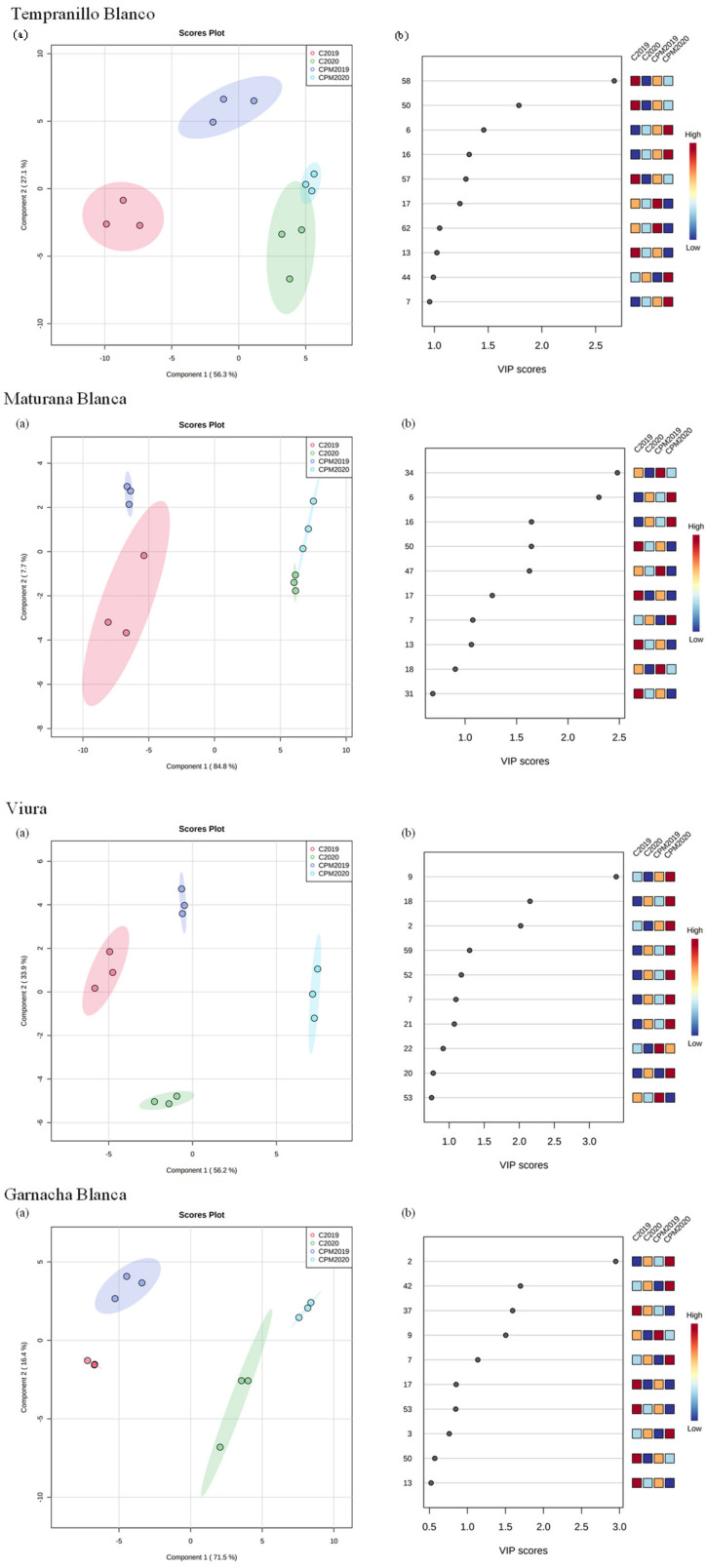
PLS-DA of the volatile fingerprint of white wines. (**a**) Score plot and (**b**) VIP scores.

Multivariate statistical analysis was carried out to provide information related to the influence of the CPM technique and vintage on the aroma compounds to enhance the complexity of wine aroma. One-way ANOVA followed by post hoc Tukey’s test at a *p*-value < 0.05 was performed to select the aroma compounds that statistically significantly differed among the studied white wines. 5-hexenyl acetate (peak n° 25), octanal (27), 3-hexen-1-ol isomer (30) and hexyl acetate (21) aroma compounds showed significant differences for ‘Tempranillo Blanco’, ‘Maturana Blanca’, ‘Viura’ and ‘Garnacha Blanca’ wines from different vintages and wines submitted to the CMP technique. Statistically different aroma compounds were submitted to PCA analysis to obtain a preliminary overview of similarities and differences between control and CPM wines. In addition, PCA can show that the CPM technique exerts a significant influence on the relative concentrations of aroma compounds, independent of vintage. To further understand the differences between control and CPM wines, a PLS-DA model was developed according to the grape variety used. As shown in Figure 3, there was a clear separation between control and CPM wines, independent of grape variety. The first two components explain 83.4%, 92.5%, 90.1% and 87.9% of the total variance for ‘Tempranillo Blanco’, ‘Maturana Blanca’, ‘Viura’ and ‘Garnacha Blanca’ wines, respectively. In addition, separation between control and CPM wines was observed for component 1 for ‘Tempranillo Blanco’ and ‘Viura’ wines, whereas for ‘Maturana Blanca’ and ‘Garnacha Blanca’ wines showed separation for component 2. Combining the VIP values higher than 1 (Figure 3b) with the loading plot revealed 10 VOCs, which were selected as aroma compound markers for the studied white wines, independent of the vintage.

For ‘Tempranillo Blanco’, the main aroma compounds that contributed to discrimination between control and CPM wines were citronellol (58), dehydro-β-cyclocitral (50), ethyl butanoate (6), 3-methyl-1-butanol (16), piperitone (57), β-phellandrene (17), octanoic acid (62), α-phellandrene (13), 2-decanol (44) and isobutanol (7), whereas for Maturana Blanca, the main discriminant aroma compounds were ethyl butanoate (6), 3-methyl-1-butanol (16), dehydro-β-cyclocitral (50), ethyl decanoate (47), β-phellandrene (17), isobutanol (7), α-phellandrene (13), ethyl hexanoate (18) and heptyl acetate (31). Regarding to Viura wines, the aroma compound markers that discriminated the control from CPM wines were isoamyl acetate (9), ethyl hexanoate (18), ethyl acetate (2), 2-phenylethyl acetate (59), diethyl succinate (52), isobutanol (7), hexyl acetate (21), m-cymene (22), 3-octanone (20) and ethyl 9-decenoate (53), whereas those for Garnacha Blanca were ethyl acetate (2), 2,3-butanediol (42), ethyl 7-octenoate (37), isoamyl acetate (9), isobutanol (7), β-phellandrene (17), ethyl 9-decenoate (53), isobutyl acetate (3), dehydro-β-cyclocitral (50) and α-phellandrene (13). Isobutanol (7) was the only aroma compound marker common to all studied white wines. In general, the mean relative concentration of these aroma compounds was higher in wines submitted to the CPM technique, except for terpenoid compounds, which presented different trends between the control and CPM wines.

**Figure 4:**
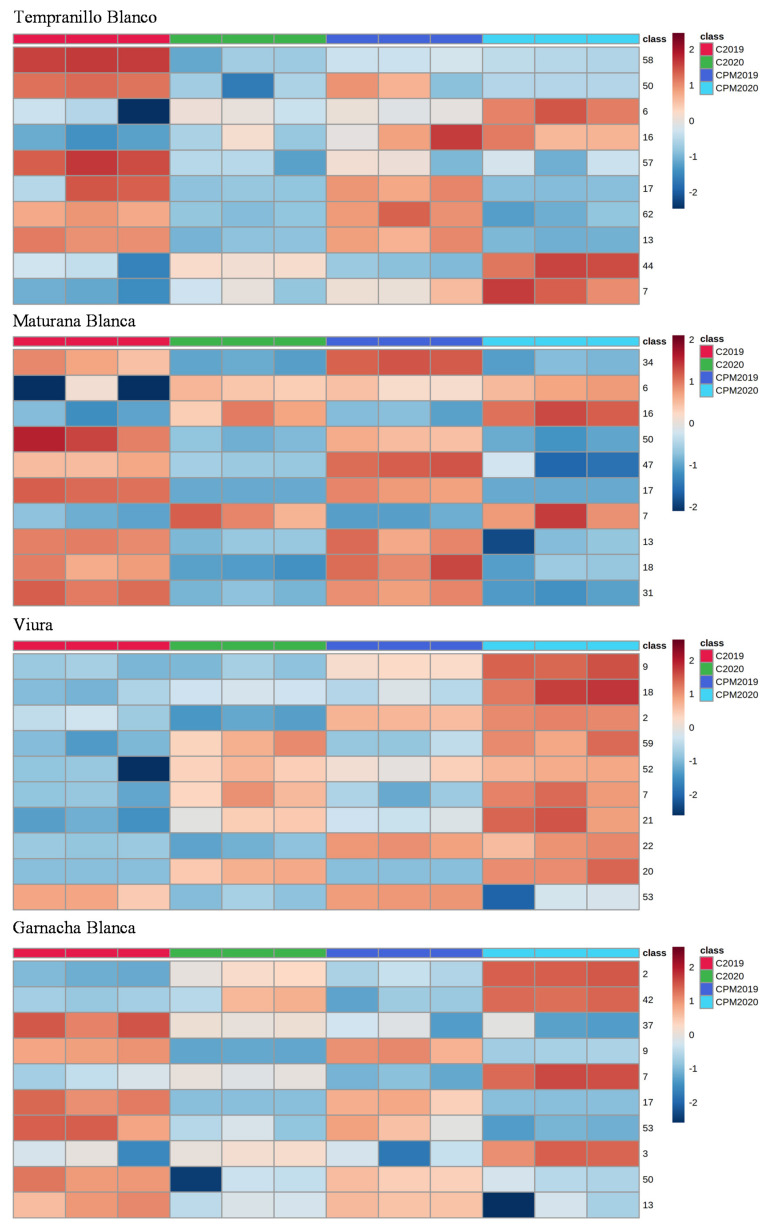
Hierarchical cluster analysis of the investigated white wines.

Figure 4 exhibits the resulting dendrogram connected to the heat map based on Pearson’s correlation, providing an intuitive visualization of the dataset, which is often employed to distinguish samples or features that are extremely high or low. A similar color tone as that in the heat map specifies the area, considering the relative concentration of the aroma compound, with VIP values higher than 1 indicating that a group of samples is comparable. The obtained result show that aroma compounds identified in the analyzed white wines present some differences between control and CPM wines, as well as between 2019 and 2020 vintages. Regarding to the vintage, the difference could be a result of climate conditions (e.g., mean annual temperature) and geographic location (e.g., longitude and latitude), which may result in an inhibition of the activity of certain odor-related enzymes, consequently influencing the aroma profile of grape and, subsequently, the wine aroma complexity [34]. Furthermore, several studies have reported that grape volatile fingerprint is associated with environmental conditions, namely altitude; soil; topography; and macro-, meso- and microclimate [35,36,37].

## 4. Conclusions

The influence of CPM treatment on the aroma compounds and sensory properties of ‘Tempranillo Blanco’, ‘Maturana Blanca’, ‘Viura’ and ‘Garnacha Blanca’ wines was evaluated during two consecutive years (2019 and 2020). A set of 62 aroma compounds, including 21 esters, 15 alcohols, 17 terpenoids, 7 carbonyl compounds and 2 acids, was identified using the HS-SPME/GC-MS methodology. The obtained data suggest that CPM treatment prior to alcoholic fermentation can modify the nutrient composition of grape must and significantly promote the formation of aroma compounds, consequently enhancing the quality of the final wine. Moreover, independent of vintage, an increase in relative the concentration of the chemical families identified in the studied white wines was verified. Alcohols and esters were the most abundant chemical families identified in the investigated white wines, and their relative concentrations in CPM wines were higher than in control wines (without CPM treatment), independent of grape variety. However, from a sensorial point of view, esters had the greatest positive contribution to the overall aroma profile of white wines with fruity and floral notes, as they are present at concentrations above their OTs (few µg/L). On the other hand, CPM treatment did not significantly influence the total relative concentration of terpenoids, and different trends were observed depending on the grape variety and vintage. In addition, independent of grape variety, some differences were observed in the relative concentration of aroma compounds identified between the two vintages, which could be a result of climate conditions and geographic location. In conclusion, CPM treatment with dry ice (solid CO_2_) represents a suitable approach to modulate the aroma compounds and enhance the aromatic quality and complexity of wines.

## Figures and Tables

**Table 1:** Enological parameters analyzed in control (C) and cold pre-fermentative maceration (CPM) white wines obtained from ‘Tempranillo Blanco’ (TB), ‘Maturana Blanca’ (MB), ‘Viura’ (V) and ‘Garnacha Blanca’ (GB) harvests in two consecutive years (2019 and 2020).

Harvest	Wine	Enological Parameters								
		Et. (% *v*/*v*)	pH	TA (g/L)	Tar.A (g/L)	MA (g/L)	P (mg/L)	YC	HCAs (CAE, %)	TPC (GAE/L)
2019	TB_C	13.1 ± 0.05	3.13 ± 0.00	8.12 ± 0.06	4.67 ± 0.07	1.89 ± 0.03	690 ± 18.9	0.09 ± 0.00	9.15 ± 0.66	10.9 ± 1.31
	TB_CPM	13.0 ± 0.05	3.27 ± 0.00	6.80 ± 0.27	2.94 ± 0.09	1.98 ± 0.01	604 ± 6.24	0.07 ± 0.00	12.3 ± 0.38	13.9 ± 0.38
	SL	ns	***	***	***	*	**	ns	**	*
2020	TB_C	13.4 ± 0.00	3.39 ± 0.01	6.11 ± 0.04	2.16 ± 0.04	2.26 ± 0.08	674 ± 6.42	0.05 ± 0.00	6.17 ± 0.05	7.15 ± 0.11
	TB_CPM	13.7 ± 0.45	3.59 ± 0.00	5.62 ± 0.02	1.60 ± 0.02	2.80 ± 0.04	881 ± 13.5	0.06 ± 0.00	6.77 ± 0.18	7.90 ± 0.23
	SL	ns	***	***	***	***	***	**	**	**
2019	MB_C	13.1 ± 0.17	3.01 ± 0.00	7.62 ± 0.07	4.91 ± 0.03	0.86 ± 0.03	569 ± 7.50	0.10 ± 0.00	3.55 ± 0.18	6.16 ± 0.16
	MB_CPM	13.1 ± 0.14	3.15 ± 0.00	6.74 ± 0.12	4.16 ± 0.13	0.68 ± 0.01	642 ± 10.4	0.13 ± 0.00	5.44 ± 0.38	8.81 ± 0.46
	SL	ns	***	***	***	**	***	**	**	***
2020	MB_C	14.4 ± 0.11	3.09 ± 0.00	6.32 ± 0.04	4.21 ±0.06	0.88 ± 0.02	572 ± 12.7	0.08 ± 0.00	2.85 ± 0.03	5.25 ± 0.02
	MB_CPM	14.1 ± 0.20	3.22 ± 0.00	5.29 ± 0.02	3.14 ± 0.05	0.98 ± 0.01	591 ± 9.84	0.09 ± 0.00	3.23 ± 0.16	5.74 ± 0.15
	SL	ns	***	***	***	**	ns	ns	*	**
2019	V_C	12.5 ± 0.14	3.16 ± 0.00	6.60 ± 0.05	4.02 ± 0.08	0.68 ± 0.06	616 ± 18.1	0.04 ± 0.00	3.88 ± 0.55	5.85 ± 0.70
	V_CPM	12.1 ± 0.17	3.28 ± 0.01	6.38 ± 0.14	3.25 ± 0.06	0.82 ± 0.05	685 ± 12.1	0.06 ± 0.00	5.22 ± 0.45	7.38 ± 0.51
	SL1	*	***	ns	***	*	**	***	*	*
2020	V_C	12.6 ± 0.41	3.21 ± 0.01	5.41 ± 0.10	3.15 ± 0.06	0.72 ± 0.02	580 ± 13.6	0.04 ± 0.00	3.62 ± 0.12	5.52 ± 0.16
	V_CPM	12.6 ± 0.20	3.33 ± 0.02	4.60 ± 0.12	2.64 ± 0.09	0.70 ± 0.10	638 ± 16.0	0.06 ± 0.00	4.30 ± 0.20	6.25 ± 0.20
	SL	ns	**	***	**	ns	**	*	**	**
2019	GB_C	13.0 ± 0.05	2.95 ± 0.00	8.36 ± 0.14	5.14 ± 0.04	0.72 ± 0.02	534 ± 8.50	0.04 ± 0.00	6.76 ± 0.83	7.24 ± 0.93
	GB_CPM	12.4 ± 0.05	3.04 ± 0.00	7.62 ± 0.05	4.59 ± 0.28	0.97 ± 0.03	640 ± 2.51	0.06 ± 0.00	8.57 ± 0.21	9.45 ± 0.15
	SL	***	***	***	*	***	***	**	*	*
2020	GB_C	13.0 ± 0.09	3.02 ± 0.01	7.20 ± 0.19	4.05 ± 0.03	0.74 ± 0.05	531 ± 0.57	0.04 ± 0.00	5.53 ± 0.10	6.05 ± 0.07
	GB_CPM	13.3 ± 0.05	3.12 ± 0.00	6.50 ± 0.14	3.46 ± 0.06	0.83 ± 0.05	600 ± 6.35	0.05 ± 0.00	6.24 ± 0.30	6.56 ± 0.31
	SL	**	***	**	***	ns	***	**	*	*

Experiments were performed in triplicate (n = 3), and results for each parameter are presented as means ± SD. SL: statistically different at the significance level: (*) *p* ≤ 0.05; (**) *p* ≤ 0.01; (***) *p* ≤ 0.001; ns, not significant. Et.—ethanol; TA—total acidity; TarA—tartaric acid; MA—malic acid; HA—hydroxycinnamic acid; YC—yellow color; TPC—total phenolic content; P—potassium; GAE—gallic acid equivalent; CAE—chlorogenic acid equivalent in % of dried material.

**Table 2:** Retention time, Kovats index (RI), odor sensory descriptors and odor threshold of aroma compounds identified in white wines analyzed by HS-SPME/GC-MS.

Peak n°	RT (min) ^1^	RI ^2^	LRI ^3^	ID ^4^	Chemical Families	Odor Sensory Descriptors ^5^	OT (µg/L) ^5^
					**Alcohols**		
5	16.86	1032	1033	S, MS, RI	1-Propanol	Alcoholic, fermented, weak fusel, musty	9000
7	19.78	1085	1085	MS, RI	Isobutanol	Ethereal winey cortex	40,000
10	23.25	1151	1151	S, MS, RI	1-Butanol	Wine-like (vinous)	150,000
16	26.72	1207	1207	S, MS, RI	3-Methyl-1-butanol	Fusel, alcoholic, pungent, ethereal, cognac, fruity	30,000
29	35.45	1357	1357	S, MS, RI	1-Hexanol	Pungent, ethereal, fruity, alcoholic	1100
30	36.22	1370	1372	MS, RI	3-Hexen-1-ol isomer	Fresh, green, grass, leaf	400
32	37.49	1390	1373	MS, RI	4-Hexen-1-ol isomer	Green herbal, musty tomato, metallic	n.d.
35	41.30	1458	1458	S, MS, RI	1-Heptanol	Musty, pungent, leafy green, fruity	200
39	44.81	1556	1547	MS, RI	2-Nonanol	Waxy, green, creamy, citrus orange	4800
42	46.67	1556	1556	MS, RI	2,3-Butanediol	Fruit	668,000
43	46.98	1613	1605	S, MS, RI	1-Octanol	Fresh orange, rose	900
44	48.31	1586	1585	MS, RI	2-Decanol	Sweet fat, floral, waxy, fruity	400
48	52.33	1634	1635	S, MS, RI	1-Nonanol	Rose, fruity	58
56	55.79	1732	1731	S, MS, RI	Methionol	Powerful sweet, soup or meat	500
61	65.24	1935	1936	S, MS, RI	Phenylethyl alcohol	Sweet, floral, fresh	14,000
					**Esters**		
2	10.41	868	870	S, MS, RI	Ethyl acetate	Caramel, sweet, fruity, buttery, pungent	7500
3	16.22	1019	1019	MS, RI	Isobutyl acetate	Sweet, fruity, ethereal, banana, tropical	1600
6	17.02	1035	1035	S, MS, RI	Ethyl butanoate	Fruity, sweet, bubblegum	20
9	21.92	1128	1128	S, MS, RI	Isoamyl acetate	Fresh, sweet, fruity	30
15	25.98	1193	1208	MS, RI	Pentyl propionate	Apple	n.d.
18	28.66	1242	1242	S, MS, RI	Ethyl hexanoate	Sweet, fruity, waxy	5
21	31.01	1281	1281	S, MS, RI	Hexyl acetate	Green, fruity, sweet, fatty, fresh	670
23	31.43	1288	1272	MS, RI	Ethyl 5-hexenoate	Fruity	n.d.
25	33.14	1317	1313	MS, RI	5-Hexenyl acetate	Mild sweet	n.d.
26	33.76	1328	1328	MS, RI	3-Hexen-1-ol acetate	Green fruity	n.d.
28	34.66	1344	1345	S, MS, RI	Ethyl heptanoate	Fruity, winey, cognac	n.d.
31	36.98	1382	1380	MS, RI	Heptyl acetate	Green, waxy, fatty, citrus, woody	n.d
34	40.51	1444	1444	S, MS, RI	Ethyl octanoate	Fruity, floral, green, leafy	2
36	41.87	1467	1468	MS, RI	Isopentyl hexanoate	Citrus, floral, oily, sweet	n.d
37	43.52	1495	1486	MS, RI	Ethyl 7-octenoate	Must, oil, fruit, pungent	n.d
40	46.12	1545	1542	S, MS, RI	Ethyl 3-hydroxybutanoate	Citrus, fruit, green, sweet	20,000
47	51.47	1625	1625	S, MS, RI	Ethyl decanoate	Sweet, fatty, nut, winey cognac	2
49	52.61	1637	1645	S, MS, RI	3-Methylbutyl octanoate	Fruity odor	125
52	53.52	1646	1642	S, MS, RI	Diethyl succinate	Fabric, fruity, flower, sweaty, potato	200,000
53	54.22	1653	1664	MS, RI	Ethyl 9-decenoate	Fruity, fatty	n.d.
59	60.84	1837	1837	S, MS, RI	2-Phenylethyl acetate	Rose, floral, fruity, sweet	250
					**Acids**		
60	62.56	1875	1876	S, MS, RI	Hexanoic acid	Sweaty, pungent, cheesy, rancid	420
62	71.33	2023	2022	S, MS, RI	Octanoic acid	Fatty, cheesy, fresh, moss	500
					**Carbonyl compounds**		
1	7.37	640	655	S, MS, RI	Acetaldehyde	Apple	80,000
20	30.12	1267	1266	S, MS, RI	3-Octanone	Musty mushroom, green vegetable	n.d.
27	33.91	1330	1329	S, MS, RI	Octanal	Fatty fruity, sweet, citrus orange	n.d
33	38.48	1406	1406	S, MS, RI	Nonanal	Fatty floral rose, waxy, citrus	15
38	44.74	1518	1518	S, MS, RI	Benzaldehyde	Burnt sugar, almond, woody	2000
51	53.21	1643	1643	MS, RI	4-Methyl-benzaldehyde	Fruity cherry, deep, phenolic	n.d.
55	55.12	1718	1718	S, MS, RI	Dodecanal	Sweet, waxy, fatty citrus, herbaceous	n.d.
					**Terpenoids**		
4	16.67	1028	1028	S, MS, RI	α-Pinene	Woody pine, camphoraceous, fresh herbal	190
8	21.21	1115	1115	MS, RI	β-Pinene	Citrus, floral, fruit, green, pine, sweet, wood	1500
11	23.66	1157	1157	MS, RI	3-Carene	Citrus fruit, orange peel	n.d.
12	24.42	1170	1170	S, MS, RI	β-Myrcene	Peppery, spicy, balsamic, plastic	14
13	24.71	1175	1175	MS, RI	α-Phellandrene	Citrus, green, black pepper	n.d.
14	25.61	1189	1189	MS, RI	α-Terpinene	Refreshing, lemony citrus	n.d.
17	27.48	1221	1220	MS, RI	β-Phellandrene	Peppery minty and slightly citrusy	n.d
19	29.66	1259	1258	MS, RI	γ-Terpinene	Sweet, citrus, tropical, lime	260
22	31.29	1286	1290	S, MS, RI	m-Cymene	Solvent, gasoline, citrus	n.d.
24	31.99	1297	1292	MS, RI	Terpinolene	Sweet-piney, oily, pleasant aroma	41
41	46.47	1552	1552	S, MS, RI	Linalool	Citrus, floral, fruity, green, muscat, sweet	15
45	48.57	1591	1606	MS, RI	Isopulegol	Minty cooling medicinal, woody, green herbal	n.d.
46	50.06	1610	1610	MS, RI	4-Terpineol	Warm peppery, mildly earthy, musty woody	110
50	52.78	1638	1642	MS, RI	Dehydro-β-cyclocitral	Saffron	n.d.
54	54.77	1711	1714	S, MS, RI	Citral	Fresh, juicy, lemon peel	n.d.
57	57.15	1759	1760	MS, RI	Piperitone	Mint	n.d.
58	57.62	1768	1768	S, MS, RI	Citronellol	Citrus, clove, floral, fresh, sweet	100

^1^ Retention time in min; ^2^ Kovats index determined using n-alkanes C_8_-C_20_ as external references; ^3^ Kovats index reported in the literature for equivalent capillary columns; ^4^ identification of volatile organic compounds (ID): S—retention time and mass spectrum consistent with that of the pure standard and with the NIST05 mass spectra electronic library; RI—Kovats index consistent with that found in the literature [16,18,26]; MS—mass spectra consistent with that from the NIST05 mass spectra electronic library; ^5^ odor threshold and odor sensory descriptors reported in the literature [4,22,23,25,27]; OT—odor threshold.

## Data Availability

Data is contained within the article or supplementary material.

## Supplementary Materials

The following supporting information can be downloaded at: https://www.mdpi.com/article/10.3390/foods12061135/s1, Table S1: Relative concentration (mg/L) and percentage of relative standard deviation (%RSD) of aroma compounds identified in the analyzed white wines; Figure S1: Total relative concentration (µg/L) of major (a) and minor (b) chemical families identified in control (C) and cold pre-fermentative maceration (CPM) white wines obtained from Tempranillo Blanco (TB), Maturana Blanca (MB), Viura (V) and Garnacha Blanca (GB) harvests in two consecutive years (2019 and 2020).
