# Supplementary material for: Influence of Cold Pre-Fermentation Maceration on the Volatilomic Pattern and Aroma of White Wines
Source: Foods. 2023 Mar 8;12(6):1135. doi: 10.3390/foods12061135 (PMC10047927; doi:10.3390/foods12061135)
Supplement: Supplementary file 1 [file foods-12-01135-s001.zip › foods-2242106-supplementary.pdf]

Influence of cold pre-fermentation maceration on the volatilomic pattern and aroma of white wines

L. Alti-Palacios [1], J. Martínez [1], José A.C. Teixeira [2], José S. Câmara[3,4,*], Rosa Perestrelo[3,*]

[1] Instituto de Ciencias de la Vid y el Vino (Gobierno de La Rioja, Universidad de La Rioja, CSIC). Finca La Gra-jera. (Ctra. de Burgos, Km. 6, 26007 Logroño, España)

[2] Departamento de Engenharia Biológica, Universidade do Minho

[3] CQM – Centro de Química da Madeira, Universidade da Madeira, Campus da Penteada, 9020-105 Funchal, Portugal

[4] Departamento de Química, Faculdade de Ciências Exatas e Engenharia, Universidade da Madeira, Campus da Penteada, 9020-105 Funchal, Portugal

* Corresponding authors: Tel.: (+351) 291705254; fax: (+351) 291705149.

E-mail address: jsc@staff.uma.pt; rmp@staff.uma.pt

Table S1. Relative concentration (mg/L) and relative standard deviation (%RSD) of aroma compounds identified in analyzed white wines.

Chemical families	2019		2020		2019		2020		2019		2020		2019		2020	
	TB_C	TB_CPM	TB_C	TB_CPM	MB_C	MB_CPM	MB_C	MB_CPM	V_C	V_CPM	V_C	V_CPM	GB_C	GB_CPM	GB_C	GB_CPM
**Alcohols**																
1-Propanol	0.06 (10)	0.11 (12)	0.18 (9)	0.14 (16)	0.10 (8)	0.10 (13)	0.25 (9)	0.22 (17)	0.04 (4)	0.04 (7)	0.14 (9)	0.18 (4)	0.09 (12)	0.07 (15)	0.15 (17)	0.21 (13)
Isobutanol	0.56 (6)	0.91 (8)	0.77 (12)	1.21 (8)	0.59 (9)	0.55 (4)	1.41 (13)	1.48 (15)	0.60 (12)	0.64 (16)	1.34 (15)	1.63 (8)	0.94 (15)	0.64 (9)	1.17 (4)	2.47 (6)
1-Butanol	0.02 (7)	0.02 (7)	0.02 (10)	0.01 (9)	0.02 (5)	0.03 (6)	0.05 (10)	0.04 (6)	0.01 (8)	0.01 (8)	0.04 (8)	0.03 (7)	0.02 (11)	0.02 (5)	0.04 (2)	0.05 (3)
3-Methyl-1-butanol	27.7 (3)	36.0 (10)	30.9 (10)	36.0 (5)	29.2 (6)	30.0 (4)	38.9 (3)	42.1 (2)	31.3 (5)	34.4 (5)	35.8 (1)	44.9 (4)	29.0 (6)	34.9 (3)	46.6 (6)	55.7 (7)
1-Hexanol	0.21 (8)	0.32 (8)	0.17 (11)	0.25 (6)	0.20 (3)	0.20 (17)	0.31 (16)	0.30 (10)	0.14 (12)	0.14 (11)	0.22 (11)	0.24 (9)	0.27 (11)	0.36 (13)	0.48 (14)	0.51 (9)
3-Hexen-1-ol isomer	-	-	-	-	0.03 (9)	0.02 (8)	0.03 (4)	0.03 (4)	0.01 (8)	0.01 (8)	0.01 (18)	0.01 (14)	0.01 (8)	0.01 (18)	0.03 (4)	0.02 (6)
4-Hexen-1-ol isomer	0.09 (3)	0.06 (9)	0.04 (17)	0.02 (10)	-	-	-	-	0.09 (6)	0.05 (18)	0.09 (8)	0.07 (13)	0.02 (7)	0.02 (15)	0.02 (3)	0.02 (17)
1-Heptanol	0.15 (11)	0.25 (4)	0.15 (8)	0.27 (4)	0.20 (6)	0.18 (6)	0.16 (8)	0.15 (6)	0.13 (11)	0.10 (12)	0.17 (6)	0.22 (13)	0.14 (13)	0.20 (4)	0.16 (9)	0.24 (3)
2-Nonanol	0.03 (3)	0.05 (6)	0.02 (3)	0.02 (5)	0.03 (13)	0.03 (13)	0.04 (9)	0.02 (8)	0.02 (13)	0.02 (18)	0.03 (15)	0.03 (17)	0.03 (10)	0.03 (9)	0.04 (5)	0.02 (7)
2,3-Butanediol	0.03 (10)	0.03 (15)	-	-	0.03 (8)	0.02 (19)	0.03 (10)	0.02 (17)	0.01 (5)	0.02 (7)	-	-	0.02 (18)	0.02 (11)	0.30 (10)	0.58 (4)
1-Octanol	0.03 (11)	0.04 (14)	0.03 (9)	0.05 (7)	0.04 (7)	0.05 (17)	0.05 (3)	0.05 (8)	0.02 (17)	0.03 (11)	0.04 (11)	0.04 (5)	0.03 (5)	0.04 (9)	0.05 (13)	0.05 (16)
2-decanol	0.03 (7)	0.01 (18)	0.06 (5)	0.17 (15)	0.03 (11)	0.04 (6)	0.10 (5)	0.11 (5)	-	-	-	-	-	-	-	-
1-Nonanol	0.02 (12)	0.04 (14)	0.02 (12)	0.03 (15)	0.03 (18)	0.03 (7)	0.02 (11)	0.03 (20)	0.01 (7)	0.02 (7)	0.02 (11)	0.02 (14)	0.03 (17)	0.02 (11)	0.03 (18)	0.03 (9)
Methionol	0.06 (17)	0.08 (3)	0.03 (10)	0.03 (5)	0.08 (14)	0.09 (20)	0.02 (8)	0.03 (13)	0.03 (11)	0.04 (5)	0.03 (16)	0.05 (16)	0.04 (18)	0.03 (12)	0.06 (10)	0.04 (6)
Phenylethyl alcohol	3.25 (8)	5.39 (8)	2.49 (16)	2.45 (13)	6.12 (15)	6.56 (7)	2.64 (15)	3.01 (3)	5.82 (7)	5.86 (12)	4.52 (18)	5.17 (7)	4.24 (15)	4.38 (10)	6.05 (10)	6.85 (8)
**Esters**																
Ethyl acetate	1.85 (7)	2.89 (3)	1.62 (6)	2.03 (17)	2.90 (8)	3.06 (16)	2.32 (16)	3.55 (17)	0.78 (16)	1.56 (3)	0.42 (9)	1.97 (2)	1.29 (9)	2.12 (12)	3.57 (11)	7.62 (2)
Isobutyl acetate	0.05 (8)	0.09 (13)	0.04 (4)	0.05 (7)	0.07 (8)	0.07 (17)	0.09 (8)	0.08 (19)	0.02 (11)	0.03 (9)	0.03 (2)	0.05 (3)	0.05 (11)	0.04 (16)	0.05 (9)	0.16 (15)
Ethyl butanoate	0.17 (14)	0.25 (6)	0.24 (15)	0.55 (11)	0.21 (6)	0.26 (17)	0.31 (10)	0.38 (11)	0.10 (17)	0.13 (14)	0.22 (11)	0.23 (8)	0.23 (17)	0.14 (11)	0.48 (2)	0.75 (6)
Isoamyl acetate	10.7 (11)	16.3 (5)	9.01 (6)	10.0 (2)	14.2 (5)	19.0 (4)	12.7 (3)	19.6 (8)	2.34 (12)	4.46 (2)	2.30 (11)	8.07 (4)	9.97 (5)	10.3 (13)	15.1 (3)	27.7 (5)
Pentyl propionate	0.02 (15)	0.02 (8)	0.01 (9)	0.01 (6)	0.01 (14)	0.02 (7)	-	-	0.01 (18)	0.01 (11)	-	-	0.02 (4)	0.01 (14)	0.02 (8)	0.02 (8)
Ethyl hexanoate	2.99 (10)	5.44 (4)	2.43 (9)	3.54 (9)	7.86 (5)	8.64 (6)	4.16 (3)	4.69 (9)	1.71 (13)	2.16 (11)	2.29 (2)	4.98 (11)	1.89 (6)	2.27 (13)	3.23 (10)	5.71 (11)
Hexyl acetate	0.49 (10)	0.86 (15)	0.22 (17)	0.33 (4)	0.77 (5)	0.88 (4)	0.36 (15)	0.50 (7)	0.02 (19)	0.06 (9)	0.09 (20)	0.17 (18)	0.37 (10)	0.35 (7)	0.41 (16)	0.84 (10)
Ethyl 5-hexenoate	0.03 (5)	0.03 (8)	0.02 (10)	0.02 (11)	0.02 (7)	0.02 (9)	-	-	0.02 (8)	0.02 (7)	-	-	0.04 (3)	0.02 (12)	0.02 (18)	0.03 (13)

5-Hexenyl acetate	0.06 (12)	0.04 (11)	0.01 (13)	0.01 (8)	0.10 (8)	0.06 (2)	0.05 (13)	0.06 (2)	0.02 (13)	0.02 (5)	0.02 (7)	0.02 (10)	0.03 (9)	0.02 (18)	0.03 (13)	0.02 (13)
3-Hexen-1-ol acetate	0.17 (8)	0.20 (11)	0.07 (12)	0.07 (9)	0.03 (5)	0.03 (17)	0.02 (15)	0.02 (14)	0.05 (1)	0.06 (14)	0.06 (16)	0.09 (14)	0.02 (9)	0.02 (8)	0.03 (17)	0.04 (5)
Ethyl heptanoate	0.02 (9)	0.02 (5)	0.02 (6)	0.01 (7)	0.05 (9)	0.07 (3)	0.03 (6)	0.02 (9)	0.02 (7)	0.03 (8)	0.02 (6)	0.02 (12)	0.02 (7)	0.01 (6)	0.02 (4)	0.02 (7)
Heptyl acetate	0.05 (9)	0.11 (10)	0.03 (9)	0.02 (8)	0.07 (6)	0.06 (6)	0.02 (6)	0.02 (7)	0.02 (3)	0.02 (6)	0.02 (5)	0.03 (2)	0.03 (3)	0.03 (7)	0.02 (8)	0.02 (12)
Ethyl octanoate	9.47 (6)	15.9 (5)	5.01 (7)	7.47 (8)	27.4 (12)	35.5 (2)	9.25 (5)	10.0 (11)	5.01 (9)	6.50 (4)	7.05 (6)	10.8 (7)	5.36 (4)	5.53 (4)	9.09 (5)	9.46 (5)
Isopentyl hexanoate	0.02 (5)	0.04 (11)	0.03 (4)	0.04 (13)	0.09 (11)	0.09 (17)	0.04 (17)	0.04 (18)	0.02 (14)	0.03 (17)	0.03 (6)	0.05 (11)	0.02 (8)	0.02 (5)	0.03 (8)	0.04 (11)
Ethyl 7-octenoate	0.30 (15)	0.22 (7)	0.06 (9)	0.04 (12)	0.13 (17)	0.11 (18)	0.06 (9)	0.05 (2)	0.11 (5)	0.07 (16)	0.04 (13)	0.07 (13)	0.27 (17)	0.05 (11)	0.07 (7)	0.06 (10)
Ethyl 3-hydroxybutanoate	0.02 (7)	0.03 (4)	0.01 (8)	0.02 (10)	0.03 (4)	0.03 (11)	0.02 (16)	0.02 (16)	0.01 (5)	0.02 (12)	0.02 (11)	0.02 (14)	0.02 (5)	0.02 (6)	0.02 (17)	0.03 (17)
Ethyl decanoate	0.40 (9)	1.43 (7)	0.11 (14)	0.22 (10)	0.86 (9)	0.41 (14)	0.24 (6)	0.44 (14)	0.21 (18)	0.31 (18)	0.12 (6)	0.31 (8)	0.32 (6)	0.43 (17)	0.29 (10)	0.36 (6)
3-Methylbutyl octanoate	0.01 (14)	0.02 (16)	0.01 (10)	0.01 (11)	0.08 (14)	0.08 (19)	0.01 (18)	0.02 (16)	0.01 (13)	0.01 (13)	0.04 (10)	0.02 (11)	0.02 (19)	0.01 (13)	0.01 (18)	0.01 (5)
Diethyl succinate	0.15 (7)	0.32 (14)	0.15 (7)	0.20 (12)	0.46 (11)	0.76 (17)	0.17 (13)	0.19 (10)	0.09 (4)	0.19 (15)	0.23 (11)	0.28 (5)	0.26 (9)	0.14 (9)	0.21 (6)	0.22 (13)
Ethyl 9-decenoate	0.35 (8)	0.31 (12)	0.40 (18)	0.57 (9)	0.70 (11)	0.31 (3)	0.50 (17)	0.95 (19)	0.53 (15)	0.62 (2)	0.14 (17)	0.26 (4)	0.78 (12)	0.58 (19)	0.38 (15)	0.28 (8)
2-Phenylethyl acetate	2.65 (12)	4.61 (12)	2.10 (9)	2.79 (14)	5.57 (5)	6.32 (3)	2.88 (8)	4.76 (6)	1.02 (11)	1.25 (11)	2.34 (15)	2.69 (10)	1.91 (4)	2.38 (11)	2.89 (11)	3.27 (15)
**Acids**																
Hexanoic acid	0.35 (13)	0.56 (7)	0.38 (12)	0.43 (14)	0.41 (5)	0.48 (9)	0.41 (12)	0.52 (4)	0.34 (7)	0.36 (12)	0.39 (9)	0.52 (9)	0.47 (11)	0.66 (8)	0.74 (12)	0.71 (14)
Octanoic acid	1.51 (6)	1.74 (13)	0.49 (5)	0.42 (18)	1.89 (10)	1.72 (7)	0.47 (6)	0.48 (13)	0.88 (6)	1.01 (4)	0.39 (17)	0.58 (18)	1.14 (12)	2.82 (16)	1.19 (3)	0.81 (19)
**Carbonyl compounds**																
Acetaldehyde	0.11 (7)	0.06 (5)	0.21 (3)	0.17 (15)	0.05 (11)	0.08 (16)	0.31 (6)	0.17 (17)	0.08 (7)	0.09 (17)	0.11 (16)	0.10 (15)	0.05 (13)	0.05 (9)	0.13 (14)	0.15 (4)
3-Octanone	-	-	-	-	0.03 (12)	0.03 (3)	0.02 (18)	0.02 (4)	-	-	0.07 (14)	0.10 (14)	0.01 (8)	0.01 (13)	0.04 (3)	0.06 (10)
Octanal	0.02 (2)	0.03 (5)	0.03 (6)	0.03 (6)	0.03 (5)	0.04 (5)	0.02 (16)	0.02 (18)	0.03 (16)	0.03 (11)	0.04 (12)	0.03 (8)	0.04 (11)	0.03 (14)	0.06 (2)	0.04 (8)
Nonanal	-	-	-	-	-	-	-	-	-	-	-	-	0.01 (4)	0.01 (15)	0.02 (9)	0.01 (10)
Benzaldehyde	0.06 (7)	0.03 (2)	0.02 (17)	0.02 (8)	0.02 (3)	0.02 (17)	0.03 (15)	0.01 (15)	0.02 (14)	0.03 (4)	0.02 (11)	0.03 (2)	0.02 (19)	0.02 (3)	0.03 (17)	0.03 (13)
4-Methyl-benzaldehyde	0.03 (17)	0.02 (11)	0.03 (6)	0.03 (5)	-	-	-	-	-	-	-	-	-	-	-	-
Dodecanal	0.04 (6)	0.06 (12)	0.03 (14)	0.02 (8)	-	-	-	-	-	-	-	-	-	-	-	-
**Terpenoids**																
α-Pinene	0.03 (10)	0.03 (7)	-	0.01 (16)	0.02 (13)	0.02 (4)	0.02 (7)	0.01 (15)	-	-	-	-	0.01 (14)	0.01 (15)	-	-

β-Pinene	-	-	-	-	-	0.02 (8)	-	-	-	-	-	-	-	-	-	-
3-Carene	0.02 (6)	0.02 (9)	0.01 (1)	0.01 (15)	0.01 (20)	0.02 (17)	0.01 (16)	0.01 (11)	-	-	-	-	-	-	-	-
β-Myrcene	0.05 (3)	0.04 (6)	0.02 (13)	0.02 (7)	0.03 (6)	0.03 (8)	0.03 (2)	0.02 (8)	0.01 (12)	0.01 (9)	0.02 (3)	0.02 (4)	0.02 (14)	0.02 (4)	0.02 (6)	0.02 (12)
α-Phellandrene	0.27 (8)	0.24 (15)	0.03 (17)	0.03 (11)	0.12 (4)	0.12 (17)	0.02 (14)	0.02 (8)	0.03 (7)	0.04 (16)	-	0.03 (7)	0.02 (15)	0.06 (4)	0.04 (12)	0.03 (16)
α-Terpinene	0.07 (15)	0.07 (11)	0.01 (11)	0.01 (12)	0.04 (8)	0.04 (14)	0.01 (13)	0.01 (9)	0.01 (20)	0.01 (14)	-	0.02 (3)	0.03 (6)	0.02 (7)	0.01 (12)	0.02 (13)
β-Phellandrene	0.99 (4)	0.66 (15)	0.05 (7)	0.04 (7)	0.34 (2)	0.27 (8)	-	-	0.07 (13)	0.07 (17)	-	-	0.19 (12)	0.12 (19)	-	-
γ-Terpinene	0.17 (8)	0.21 (10)	0.02 (15)	0.02 (6)	0.10 (11)	0.08 (6)	0.03 (5)	0.03 (6)	0.02 (6)	0.03 (8)	0.02 (16)	0.02 (12)	0.05 (6)	0.03 (10)	0.02 (3)	0.02 (9)
m-Cymene	0.10 (8)	0.13 (18)	0.03 (16)	0.02 (16)	0.06 (9)	0.05 (17)	0.02 (10)	0.04 (13)	0.03 (3)	0.11 (5)	0.03 (16)	0.11 (14)	0.05 (5)	0.05 (9)	0.03 (14)	0.01 (8)
Terpinolene	0.03 (6)	0.04 (15)	0.03 (4)	0.03 (9)	0.04 (18)	0.03 (16)	0.04 (16)	0.05 (11)	0.02 (8)	0.03 (4)	0.04 (11)	0.05 (12)	0.02 (13)	0.03 (11)	0.05 (5)	0.07 (4)
Linalool	0.10 (7)	0.04 (7)	0.05 (7)	0.05 (3)	0.03 (2)	0.03 (7)	0.07 (8)	0.05 (16)	0.01 (2)	0.02 (15)	0.12 (5)	0.15 (9)	0.02 (15)	0.02 (12)	0.03 (4)	0.04 (3)
Isopulegol	0.03 (4)	0.02 (16)	0.03 (4)	0.02 (7)	-	-	-	-	-	-	-	-	-	-	-	-
4-Terpineol	0.05 (2)	0.02 (4)	0.01 (8)	0.01 (12)	0.02 (17)	0.01 (15)	0.02 (13)	0.01 (11)	0.01 (10)	0.01 (16)	0.02 (8)	0.01 (16)	0.01 (18)	0.01 (6)	0.01 (12)	0.02 (18)
Dehydro-β-cyclocitral	1.82 (3)	1.35 (15)	0.02 (9)	0.26 (1)	1.07 (15)	0.73 (5)	0.32 (7)	0.27 (9)	0.44 (12)	0.33 (8)	0.20 (6)	0.30 (7)	0.66 (9)	0.47 (7)	0.29 (13)	0.26 (14)
Citral	0.06 (16)	0.07 (12)	0.06 (10)	0.09 (8)	-	-	-	-	-	-	-	-	-	-	-	-
Piperitone	0.18 (12)	0.03 (8)	0.01 (5)	0.02 (11)	-	-	-	-	0.03 (17)	0.04 (8)	0.03 (18)	0.06 (6)	0.05 (9)	0.04 (13)	0.06 (10)	0.05 (6)
Citronellol	0.12 (12)	0.07 (10)	0.02 (8)	0.04 (18)	0.05 (11)	0.06 (13)	0.03 (17)	0.03 (10)	0.03 (6)	0.03 (16)	0.03 (15)	0.06 (16)	0.03 (5)	0.02 (14)	0.04 (15)	0.03 (6)

Abbreviation: TB – Tempranillo Blanco; MB – Maturana Blanca; V – Viura; GB – Garnacha Blanca; C – control; CPM – cold pre-fermentative maceration.

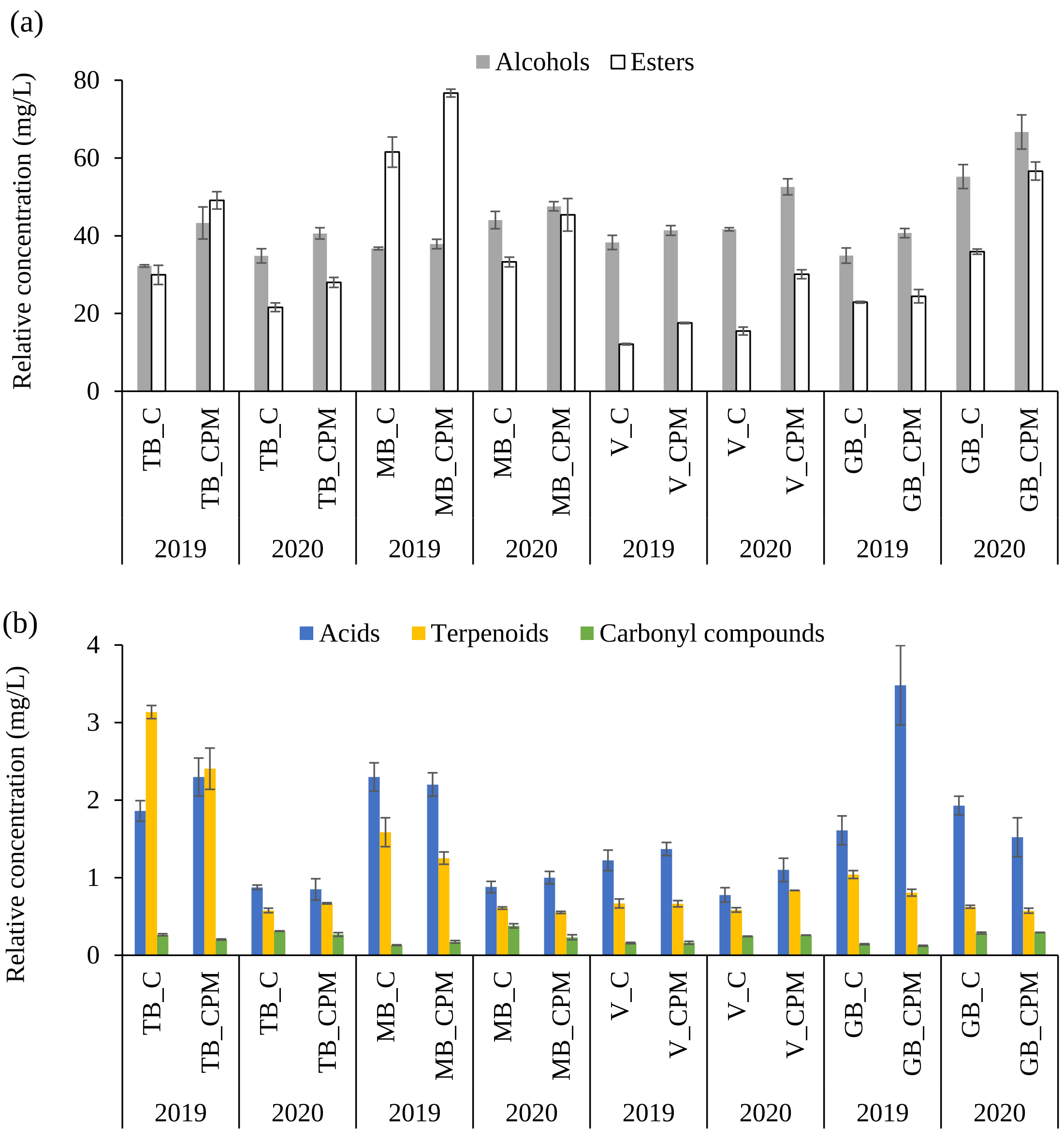

Figure S1. Total relative concentration (µg/L) of major (a) and minor (b) chemical families identified in control (C) and cold pre-fermentative maceration (CPM) white wines obtained from Tempranillo Blanco (TB). Maturana Blanca (MB). Viura (V) and Garnacha Blanca (GB) harvest in two consecutive years (2019 and 2020).
